# Pemigatinib for previously treated metastatic or unresectable central nervous system tumors with fibroblast growth factor receptor mutations or rearrangements: FIGHT-207 results

**DOI:** 10.1093/oncolo/oyaf272

**Published:** 2025-10-16

**Authors:** Iben Spanggaard, Marc Matrana, Caio Rocha Lima, Amit Mahipal, Maria Vieito, Alice Hervieu, Lipika Goyal, Jordi Rodón, Maria Luisa Veronese, Natalia Oliveira, Xin Li, Michael Schaffer, Santosh Kesari

**Affiliations:** Department of Oncology, Rigshospitalet Copenhagen University Hospital, Copenhagen 2100, Denmark; Ochsner Cancer Institute, Ochsner Clinic Foundation, New Orleans, LA 70121, United States; Department of Hematology and Oncology, Wake Forest University School of Medicine—Atrium Health, Winston-Salem, NC 27157, United States; Department of Oncology, Mayo Clinic, Rochester, MN 55905, United States; Seidman Cancer Center, Case Western Reserve University, Cleveland, OH 44106, United States; Department of Medical Oncology, Vall d’Hebron University Hospital, Barcelona 08035, Spain; Department of Medical Oncology, Centre Georges-François Leclerc, Dijon 21000, France; Mass General Cancer Center, Harvard Medical School, Boston, MA 02114, United States; Stanford Cancer Center, Stanford School of Medicine, Stanford, CA 94305, United States; MD Anderson Cancer Center, The University of Texas, Houston, TX 77030, United States; Incyte International Biosciences Sàrl, Morges 1110, Switzerland; Incyte International Biosciences Sàrl, Morges 1110, Switzerland; Incyte Corporation, Wilmington, DE 19803, United States; Incyte Corporation, Wilmington, DE 19803, United States; Providence Southern California Research Clinical Institute, Saint John’s Cancer Institute, Pacific Neuroscience Institute, Los Angeles, CA 90404, United States

**Keywords:** pemigatinib, central nervous system tumors, fibroblast growth factor receptors, glioblastoma

## Abstract

Central nervous system (CNS) tumors often harbor alterations in genes regulating key cellular pathways, including fibroblast growth factor receptor (*FGFR*) genes. Here, we report the efficacy and safety of treatment with pemigatinib, an oral, potent, selective FGFR1-3 inhibitor, in patients with advanced *FGFR*-altered CNS tumors. FIGHT-207 was a single-arm, open-label, phase 2 study of pemigatinib in patients with advanced solid tumors harboring *FGFR* fusions/rearrangements or other mutations. Patients received pemigatinib 13.5 mg once daily until disease progression or unacceptable toxicity. Endpoints included tumor response and safety. Of the 13 patients with CNS tumors in FIGHT-207, 10 had glioblastoma. Fibroblast growth factor receptor alterations were *FGFR3-TACC3* fusions (*n* = 9), *FGFR1* K656E mutations (*n* = 2), *FGFR1* N546K mutation (*n* = 1), and *FGFR1-MITF* fusion (*n* = 1). Three patients (23%) displayed objective responses (1 complete, 2 partial). Safety was consistent with the overall FIGHT-207 population. Pemigatinib had antitumor activity and a manageable safety profile in patients with CNS tumors.

## Brief communication

Pemigatinib, an oral, potent, selective fibroblast growth factor receptor (*FGFR*)*1-3* inhibitor, was evaluated in FIGHT-207, a phase 2 basket study in patients with previously treated, unresectable or metastatic solid tumors harboring *FGFR* fusions/rearrangements, activating single nucleotide variants (SNV) excluding kinase domain mutations, or variants of unknown significance (VUS). Patients received pemigatinib at starting dose of 13.5 mg once daily until disease progression or unacceptable toxicity. The FIGHT-207 results were recently published.^1^ Pemigatinib demonstrated antitumor activity in central nervous system (CNS), gynecologic, and pancreatic tumors, in addition to cholangiocarcinoma.

Molecular profiling of CNS tumor tissue has revealed genomic alterations, including oncogenic *FGFR* alterations, that may be appropriate for treatment with targeted therapies. The most prevalent *FGFR* alterations in gliomas (the most common malignant CNS tumors^2^) are *FGFR3* rearrangements and *FGFR1* mutations.^3^ Pemigatinib is approved to treat cholangiocarcinomas with *FGFR2* fusions or alterations and myeloid/lymphoid neoplasms with *FGFR1* rearrangement.^4^ The antitumor activity of pemigatinib for challenging-to-treat CNS tumors was previously unknown. Here, we highlight the efficacy, safety, and translational findings from the subset of patients with CNS tumors enrolled in FIGHT-207.

Of the 107 efficacy-evaluable patients enrolled in FIGHT-207, 13 patients had unresectable or metastatic CNS tumors. Of these, one patient with no prior treatment was permitted to enroll in FIGHT-207 because no treatment deemed of benefit existed for the patient’s tumor type. Ten patients harbored *FGFR* fusions and rearrangements and 3 had *FGFR* kinase domain mutations or VUS. Median (range) age was 60.0 (43-71) years, 61.5% were women, and 76.9% were White. Central nervous system tumors were classified as glioblastoma (GBM; *n* = 10, 76.9%), polymorphous low-grade neuroepithelial tumor of the young (PLNTY; *n* = 1, 7.7%), diffuse astrocytoma grade 2 (*n* = 1, 7.7%), and low-grade pediatric type glioma (*n* = 1, 7.7%). Most patients had prior radiation (*n* = 10), surgery (*n* = 10), and systemic therapies (*n* = 9). Fibroblast growth factor receptor alterations included *FGFR3-TACC3* fusions (*n* = 9), *FGFR1* K656E mutations (*n* = 2), *FGFR1* N546K mutation (*n* = 1), and *FGFR1-MITF* fusion (*n* = 1).

**Figure 1.**
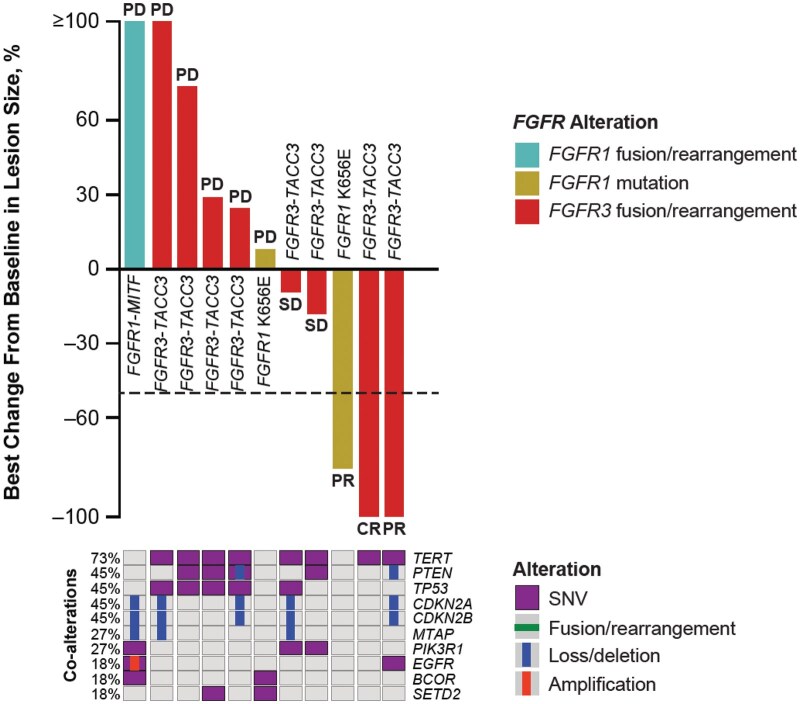
Best percent change from baseline, best overall response, and co-alterations at baseline in patients with CNS tumors. Best percent change from baseline by RANO for all evaluable patients with CNS tumors; best overall response by IRC indicated where evaluable. Two patients were excluded: No target lesion measurement was available (*n* = 1), and no IRC response assessments were available (*n* = 1). Baseline co-alterations are from local reports, central FMI, and baseline Predicine ctDNA data. The dashed line indicates a criterion for PR per RANO (≥50% decrease in target lesion size). CNS, central nervous system; CR, complete response; ctDNA, circulating tumor DNA; FGFR, fibroblast growth factor receptor; FMI, Foundation Medicine, Inc.; IRC, independent review committee; PD, progressive disease; PR, partial response; RANO, Response Assessment in Neuro-Oncology; RECIST, Response Evaluation Criteria in Solid Tumors; SD, stable disease; SNV, single nucleotide variant. One patient had 100% tumor shrinkage (CR) for the target lesion, but had SD for a non-target lesion, and so was classified as having PR.

**Figure 2.**
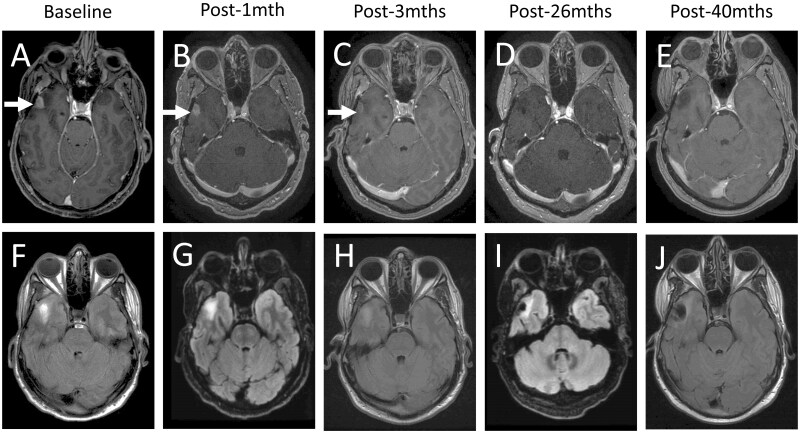
Brain MRIs showing durable complete response for patient. Patient with recurrent right temporal glioblastoma failing chemoradiation with 7 cycles of adjuvant temozolomide with Optune^®^ device, then re-resected gross total resection confirmed active glioblastoma, and subsequently progressed after 2 cycles of lomustine before starting pemigatinib on study. A-E: axial post-gadolinium MRI T1 sequences showing resolution of enhancing temporal mass over time; F-J: axial MRI T2/FLAIR sequences showing the resolution of edema over time.

Five patients (38.5%) experienced a reduction from baseline in target lesion size with pemigatinib treatment (Figure 1). Three (23%) patients had objective response per Response Assessment in Neuro-Oncology (complete response [CR], *n* = 1; partial response [PR], *n* = 2) and 3 (23%) patients had stable disease (SD; for 1 patient with SD, no target lesion measurement was available). The patient with CR had unmethylated GBM with an *FGFR3-TACC3* fusion. Time to response was 4.4 months, and the duration of response was 15.9 months (Figure 2). No patient deaths were observed at the time of database lock and overall survival (OS) was censored at 20.2 months. Analysis of tumor tissue from the patient with CR indicated an unmethylated *MGMT* promoter. One patient with PR had GBM with a methylated *MGMT* promoter and an *FGFR3-TACC3* fusion. The second patient had a diffuse astrocytoma grade 2, with an *FGFR1* K656E mutation. Two of the 3 patients with SD harbored *FGFR3-TACC3* fusions. In these 2 patients, progression-free survival (PFS) was 3.7 and 4.1 months, and OS was 6.1 and 13.3 months. The third patient with SD, who had an *FGFR1* N546K mutation and had not received prior systemic therapy, experienced PFS and OS of 6.2 and 6.3 months, respectively. This patient was initially diagnosed with pediatric low-grade glioma and had received surgery as the only prior therapy. It should be noted that harboring a potentially actionable genetic alteration does not guarantee targeted treatment efficacy, and the best option for these patients at primary diagnosis may be the current standard of care.

In our analysis, most patients had *FGFR3-TACC3* fusions (*n* = 9; 8 patients with GBM, 1 patient with PLNTY), with the remaining patients exhibiting *FGFR1* mutations (K656E, *n* = 2, 1 patient with GBM, 1 patient with diffuse astrocytoma grade 2; N546K, *n* = 1, patient had other glioma) and an *FGFR1-MITF* fusion (*n* = 1, patient had GBM). *TERT* SNVs were the most common co-alterations in this patient population with a frequency of 73% and 90% in patients overall and with *FGFR3-TACC3* fusions, respectively. *TERT* mutations have been reported in nearly 80% of the patients with GBM.^5^ The incidence of *TERT* co-alterations in our study was generally consistent with the literature. In our study, the extent to which the identified co-alterations impacted clinical outcomes in patients with CNS tumors with *FGFR* alterations remains unclear.

Full safety data for patients in FIGHT-207 have been previously published.^1^ The safety of pemigatinib in patients with CNS tumors was consistent with the overall FIGHT-207 population. All patients in FIGHT-207 and in the CNS-tumor subgroup experienced treatment-emergent adverse events (TEAEs); hyperphosphatemia was the most common TEAE (FIGHT-207 overall, 83.8%; FIGHT-207 CNS, 84.6%). In the CNS-tumor subgroup, TEAEs were grade ≥3 in 76.9% of patients and led to dose reduction, treatment interruption, or discontinuation in 38.5%, 76.9%, and 15.4%, respectively. The most common grade ≥3 TEAEs related to pemigatinib were nail changes/disorders (*n* = 3) and stomatitis (*n* = 2). Treatment-emergent adverse events leading to pemigatinib discontinuation were spinal cord compression (*n* = 1) and pancreatitis (*n* = 1). One patient had a fatal TEAE (sepsis), which was not considered to be related to pemigatinib.

In summary, treatment with pemigatinib demonstrated antitumor activity and manageable TEAEs in patients with CNS tumors harboring *FGFR* fusions and kinase domain mutations. These findings warrant confirmation in the ongoing phase 2 FIGHT-209 study (NCT05267106) of pemigatinib in patients with recurrent GBM and other CNS tumors with activating *FGFR1-3* mutations or fusions/rearrangements.^6^ The findings from FIGHT-209 will provide additional data concerning the use of pemigatinib in patients with CNS tumors and susceptible *FGFR* alterations.

## Data Availability

Incyte Corporation (Wilmington, DE, United States) is committed to data sharing that advances science and medicine while protecting patient privacy. Qualified external scientific researchers may request anonymized datasets owned by Incyte for the purpose of conducting legitimate scientific research. Researchers may request anonymized datasets from any interventional study (except phase 1 studies) for which the product and indication have been approved on or after January 1, 2020 in at least one major market (eg, United States, European Union, and Japan). Data will be available for request after the primary publication or 2 years after the study has ended. Information on Incyte’s clinical trial data sharing policy and instructions for submitting clinical trial data requests are available at: https://www.incyte.com/Portals/0/Assets/Compliance%20and%20Transparency/clinical-trial-data-sharing.pdf? ver=2020-05-21-132838-960.
